# Identification of a new allele of *catp-4*

**DOI:** 10.17912/micropub.biology.001523

**Published:** 2025-03-24

**Authors:** Anushree Gurjar, Amber R Krauchunas

**Affiliations:** 1 Biological Sciences, University of Delaware, Newark, Delaware, United States

## Abstract

CATP-4 is a subunit of a Na ^+^ /K ^+^ -ATPase important for sperm motility and fertility. Here, we report the characterization of a new allele of *catp-4* isolated from a forward genetic screen for sterile mutants. The *catp-4 ( as46 )* allele has a missense mutation that substitutes a highly conserved Glycine which lies close to an ATP binding site. This mutation results in a loss-of-function phenotype comparable to that of the *catp-4* null alleles, emphasizing the importance of this residue for CATP-4 function. This is for the first time that a phenotype has been reported for a single missense mutation in the *catp-4* gene.

**Figure 1. Phenotypic characterization of as46 and its causative mutation:**
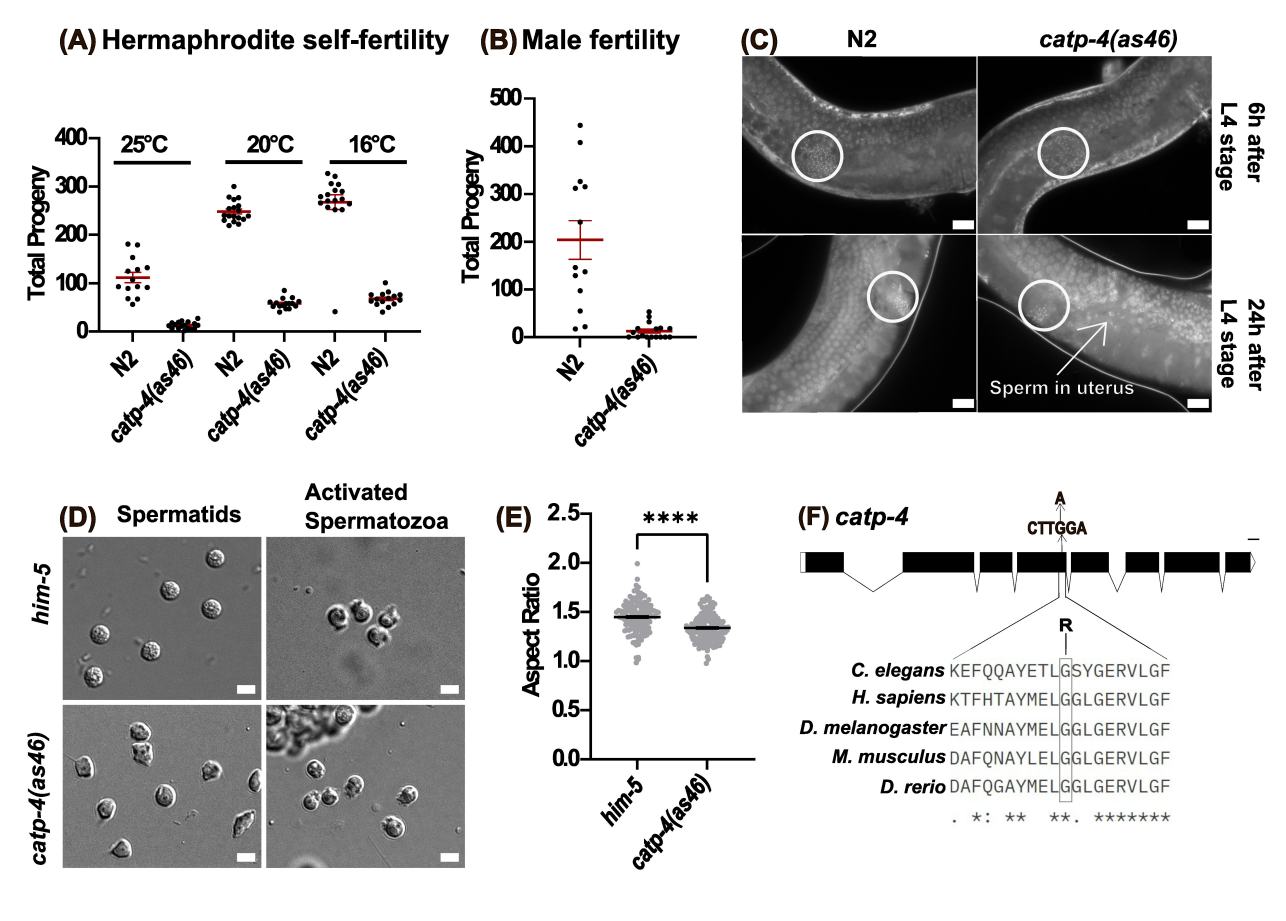
A. Hermaphrodite self-fertility is significantly reduced compared to wild-type N2 hermaphrodites (p<0.0001). B. *catp-4 ( as46 ); him-5* male fertility is significantly reduced compared to wild-type N2 males when mated to *fog-2 ( oz40 )* hermaphrodites (p<0.0001). Each dot on the graph represents the total number of progeny produced by one individual hermaphrodite (n≥10). Error bars represent standard error (SEM). C. DAPI staining of *catp-4 ( as46 )* and wild-type N2 hermaphrodites at L4+6 hrs L4+24 hrs. The spermathecae are marked by circles. Presence of sperm in the uterus (indicated by an arrow) is only observed in *catp-4 ( as46 )* hermaphrodites. Scale bar = 20µm D. Morphology of spermatids and activated spermatozoa from *him-5 ( e1490 )* control males and *catp-4 ( as46 ); him-5 ( e1490 )* males. Scale bar = 10µm E. Aspect ratio comparison of *him-5 ( e1490 )* sperm and *catp-4 ( as46 ); him-5 ( e1490 )* sperm (n=140). Error bars represent standard error (SEM). F. Schematic of the *catp-4* genomic locus indicating the location of the *as46* mutation in the fourth exon. Below is an alignment of *C. elegans* CATP-4 with Na ^+^ /K ^+^ -ATPases from other organisms. The conserved glycine residue that is changed from glycine (G) to arginine (R) in the *catp-4 ( as46 )* mutant is marked with a black rectangle.

## Description

We report the identification of a new allele of *catp-4* , which we refer to as *catp-4 ( as46 )* . CATP-4 is a subunit of a Na+/K+-ATPase required for sperm activation, sperm motility, and fertility in *C. elegans* (Wang et al., 2021). The *as46* allele was isolated from a forward genetics EMS (ethyl methanesulfonate) screen for sterile mutants, similar to the one described in Singaravelu et al., 2015. Brood sizing assays show that both *catp-4 ( as46 )* hermaphrodites and *catp-4 ( as46 )* males have significantly reduced fertility (p<0.0001) compared to wild-type N2 ( Figure 1A and 1B).

To determine the reason for reduced fertility of *catp-4 ( as46 )* hermaphrodites we examined whether sperm were produced and correctly localized within the spermatheca. Sperm nuclei appear as small bright punctae by DAPI staining. It was observed that at 6 hours after the L4 larval stage, there was no visual difference between the complement of sperm in unmated *catp-4 ( as46 )* hermaphrodites and unmated N2 hermaphrodites ( Figure 1C ). At this time point spermatogenesis should be complete but the first ovulation has not yet taken place. However, at 24 hours after the L4 stage, when multiple ovulations have occurred, the number of sperm in the spermathecae for *catp-4 ( as46 )* hermaphrodites appeared noticeably reduced relative to wild type. We also observed sperm in the uterus which was not observed for wild-type N2 hermaphrodites ( Figure 1C ). This loss of sperm is indicative of sperm migration and motility defects. As an egg moves from the spermatheca to the uterus sperm are pushed out into the uterus and must crawl back into the spermatheca for the opportunity to fertilize a subsequent egg (L'Hernault, 2006; Ward & Carrel, 1979). If they are unable to migrate back to the spermatheca, the sperm are rapidly lost as ovulations continue. Consistent with our observation of rapid sperm loss from the spermathecae, spermatids dissected from *catp-4 ( as46 )* mutant males suffer from severe morphological defects ( Figure 1D ). Instead of having a normal spherical morphology, the spermatids are angular and distorted. In addition, when these spermatids are exposed to Pronase to trigger sperm activation *in vitro* , they form pseudopods that are shorter than wild-type controls ( Figure 1D ). To quantify the relative length of the pseudopods we measured the aspect ratio for the sperm, by dividing the sperm length (cell body and pseudopod) by the sperm width (cell body). A ratio closer to 1 indicates shorter pseudopods. The aspect ratio is significantly smaller (p < 0.0001) for *catp-4 ( as46 ); him-5* sperm (1.336 ± 0.01188, n = 140 ) compared to *him-5* sperm (1.448 ± 0.01381, n = 140 ) ( Figure 1E ). Taken together our data indicate that the *as46* mutation causes defects in sperm activation and motility.

Whole genome sequencing identified that the *as46* allele is a missense point mutation in exon 4 of *C01G12.8 ,* aka *catp-4* . The missense mutation causes an amino acid change from glycine (G) to arginine (R) at the 532nd amino acid position ( Figure 1E ). The mutation was confirmed by Sanger sequencing. The phenotype we observe for *as46* matches the phenotype of *catp-4* null alleles already reported (Wang et al., 2021).

To verify that the observed phenotype is due to the missense mutation in the *catp-4* gene, we performed a complementation test at 25°C where the *as46* phenotype is the most severe. If two strains carry a mutation in the same gene, then upon crossing a heterozygous worm from one strain to a homozygous mutant worm from the other strain, we expect 50% of the F1 progeny to show the mutant phenotype. When heterozygous *catp-4 ( ok2056 )* /+ males were crossed with homozygous *as46* hermaphrodites, 45.8% of F1 hermaphrodites showed the *catp-4* phenotype of reduced progeny production (11 out of 24). Furthermore when heterozygous *as46* /+ males were crossed with homozygous *catp-4 ( ok2056 )* hermaphrodites, 54.2% (13 out of 24) of F1 hermaphrodites showed the *catp-4* phenotype. Therefore, we conclude that the two alleles fail to complement and *as46* is an allele of *catp-4* .

EMBL-EBI MUSCLE was used to align the *C. elegans* CATP-4 protein with Na+/K+-ATPases from other organisms. We found that the *as46* mutation changes a highly conserved glycine ( Figure 1F ). This conserved glycine residue must be critical for CATP-4 protein function as substitution of an arginine at this site recapitulated the phenotype of a complete loss of function of *catp-4* . The glycine lies between two ATP binding sites, one of which is only four amino acids away. We hypothesize that since the substitution of glycine with arginine is in close proximity to an ATP binding site, it potentially affects the structure of the ATP binding site, and ultimately the protein's function. However, further experiments will be needed to confirm this hypothesis.

## Methods

*C. elegans* maintenance

All *C. elegans* strains were maintained according to (Brenner, 1974). Worms were cultured on MYOB plates seeded with OP50 *E. coli* . Strains were maintained at 16°C. For experiments, adult worms were placed at 16°C for 2 days and then their eggs/L1 progeny were shifted to 25°C. All experiments were carried out at 25°C unless stated otherwise.

Hermaphrodite self-fertility and male fertility

Fertility was determined by measuring the brood size of individual animals. To determine hermaphrodite self-fertility, L4 hermaphrodites were selected and put on individual plates. These hermaphrodites were transferred to new plates every 24 hours until they stopped laying any embryos. To determine male fertility, males were put on individual plates with *fog-2 ( oz40 )* L4 hermaphrodites in a 4:1 ratio. *fog-2 ( oz40 )* hermaphrodites do not produce sperm and therefore all progeny are known to be sired by the males. After 24 hours, the males were removed and the hermaphrodites were transferred daily to new plates until no new embryos were seen on the plates. For both hermaphrodite self-fertility and male fertility assays, once the hermaphrodite was removed from the plate the progeny were grown at 20°C and counted 3 days later. Hermaphrodites that disappeared or died during the experiment were excluded from the analysis.

DAPI staining

Hermaphrodites were selected at the L4 stage and aged for either 6 hours or 24 hours. The hermaphrodites were fixed with ethanol and stained with DAPI (1 mg/ml) (Clarke et al., 2018). Images were taken using a Zeiss Observer D.1 with a 40X objective and captured with an AxioCAM 503 camera.

Spermatid morphology and sperm activation

Young adult males were separated from hermaphrodites for approximately 24 hours prior to the experiment. To determine spermatid morphology and sperm activation, males were dissected in Sperm Media (50 mM HEPES, 25 mM KCl, 45 mM NaCl, 1 mM MgSO _4_ , 5 mM CaCl _2_ , 10 mM Dextrose, pH 7.8) modified from (L'Hernault SW & Roberts TM, 1995) or Sperm Media with Pronase (200 µg/ml), respectively. DIC images were taken using a Zeiss Observer D.1 with a 40X objective and captured with an AxioCAM 503 camera. The aspect ratio was calculated by dividing the sperm's total length of the cell body and the pseudopod by the width of the cell body modified from (Hansen et al., 2015).

Whole genome sequencing

The one step whole genome sequencing and SNP mapping strategy for mutant identification was employed wherein *catp-4 ( as46 )* hermaphrodites were crossed with Hawaiian strain CB4856 males (Doitsidou et al., 2010). The F2 progeny (approximately 90 worms) that exhibited the *as46* phenotype were lysed and prepped as in (Jaramillo-Lambert et al., 2015). Sequencing was done at the University of Delaware Sequencing and Genotyping Center using an Illumina sequencer. The data analysis was done using Mimodd v0.1.9 ( https://mimodd.readthedocs.io/en/latest/nacreousmap.html ).

Sanger sequencing

An approximately 5.5 kb genomic sequence was amplified from lysed N2 and *catp-4* ( *as46* ) worms. For Sanger sequencing, we used a forward sequencing primer (5'-AGATACTGCGAGATGATTCG-3') 261 bp upstream of the site of the mutation. The Sanger sequencing results for N2 and *catp-4 ( as46 )* were trimmed for low quality bases and aligned to the *catp-4* gene sequence from Wormbase (Sternberg et al., 2024).

Complementation test

In order to create trans-heterozygotes we needed males carrying an allele of *catp-4* . Therefore, we crossed *catp-4 ( ok2056 )* and *catp-4 ( as46 )* L4 hermaphrodites to young adult N2 males at 20°C. The heterozygous *catp-4* ( *ok2056* ) male progeny and heterozygous *catp-4 ( as46 )* male progeny were crossed into *catp-4 ( as46 )* L4 hermaphrodites and *catp-4* ( *ok2056* ) L4 hermaphrodites respectively at 25°C to perform the complementation test. Their F1 progeny were singled out to determine whether they had reduced fertility.

Statistical analysis

t-tests and statistics were performed using GraphPad Prism version 9.1.2 for Windows, GraphPad Software, Boston, Massachusetts USA, www.graphpad.com

Software

Figure 1C and Figure 1D were assembled using Fiji (version 1.54p) (Schindelin et al., 2012) plugin Quickfigures (version 2023.2) (Mazo, 2021). The aspect ratio was measured using the line tool in Fiji. The MUSCLE multiple sequence alignment was executed using the job dispatcher on the EMBL EBI website (Madeira et al., 2024).

## Reagents

**Table:** 

Strain	Genotype	Available from
N2	Wild type	CGC
DR466	*him-5 ( e1490 )* V	CGC
VC1649	*C01G12.8 ( ok2056 )* II	CGC
AD378	*catp-4 ( as46 )* II	Krauchunas Lab
ARK3	*catp-4 ( as46 )* II; *him-5 ( e1490 )* V	Krauchunas Lab
DG4915	*fog-2 ( oz40 ); his-72 ( uge30 )[gfp:: his-72 ] *	Jaramillo-Lambert Lab, University of Delaware
